# Retinal Photoreceptor Expresses Toll-Like Receptors (TLRs) and Elicits Innate Responses Following TLR Ligand and Bacterial Challenge

**DOI:** 10.1371/journal.pone.0119541

**Published:** 2015-03-13

**Authors:** Pawan Kumar Singh, Ashok Kumar

**Affiliations:** 1 Kresge Eye Institute, Wayne State University School of Medicine, Detroit, Michigan, United States of America; 2 Department of Anatomy and Cell Biology, Wayne State University School of Medicine, Detroit, Michigan, United States of America; UC Irvine Medical Center, UNITED STATES

## Abstract

Toll-like receptors (TLRs) play an important role in host defense against microbial pathogens. Our previous studies have shown that TLRs are expressed on various retinal cells (Microglia and Müller glia) and orchestrate retinal innate responses in bacterial endophthalmitis. In this study, we used a well-characterized mouse cone photoreceptor cell line (661W); and demonstrated that these cells express all known TLRs. Although the stimulation of 661W cells with TLR ligands (Pam3Cys, PolyI:C, LPS, Flagellin, Poly DT, and ODN) did not alter TLR expression, downstream TLR-signaling pathways (NF-κB, p38, and ERK) are activated. Moreover, TLR-activated 661W cells secreted significant amounts of inflammatory mediators (IL-6, IL-1β, MIP-2, and KC) in their culture supernatant, as assessed by ELISA. A similar trend was observed in 661W cells challenged with live bacteria (*Staphylococcus aureus*). Interestingly, the neutralization of TLR2, a major receptor for *S*. *aureus* recognition, did not significantly attenuate bacterial-induced inflammatory mediators, suggesting the existence of TLR2-independent mechanisms in photoreceptor cells. Together, these results indicate that photoreceptors constitutively express functional TLRs and possess the ability to initiate innate responses following pathogen challenge, implicating their role in retinal innate immunity.

## Introduction

TLR signaling is one of the best studied and well characterized pathways to initiate host immune defense mechanisms against invading microbes in both humans and animals [1,2,3,4]. TLRs are essential mediators of innate and adaptive immunity and respond to a wide variety of microbial ligands and the “danger signals” produced by the host during infection [2,3,5]. TLRs are highly expressed on professional immune cells that have pathogen surveillance activity [4]. However, a wide variety of non-hematopoietic cells, such as neurons, glia, and several types of epithelial cells, also express TLRs, suggesting additional physiological functions for TLRs [1]. In the eye, TLRs have been reported to be expressed by a variety of cell types; however, their expression pattern (cell surface vs. intracellular) may differ for one or more individual TLRs [2,3,6]. For instances, the cornea and conjunctiva express most of the TLRs, while TLR4 is the only known to be expressed by the uvea and sclera [7,8]. Similarly, there are differences in the expression of TLRs at the transcript and protein levels from different tissues; for example, some cells express only transcripts, while others produce functional TLRs [2,6]. A detailed study of this variability in the expression of individual TLRs in different parts of the eye reveals some sort of strategic evolution which seems to have contributed to the immune privileged state of the eye [2]. Among the ocular cell types, almost all cells have been investigated to some extent for the expression of TLRs. However, the expression of TLRs by photoreceptor cells has not been fully investigated.

The rod and cone photoreceptors are the light sensing cells and constitute the major cell population in the retina. Our recent studies have shown that the loss of vision (decline in ERG response) in an experimental model of bacterial endophthalmitis is accompanied by the death of retinal cells, including photoreceptors. The induction of photoreceptor cell death could be due to the increased inflammatory milieu or the direct action of the bacterial pathogen [9]. How photoreceptors contribute to the retinal innate response in endophthalmitis has not been investigated. Interestingly, a recent study by Tu *et*. *al*. demonstrated that both primary and 661W photoreceptor cell lines constitutively express TLR4 and secret inflammatory mediators following challenge with LPS [10]. To determine whether photoreceptor cells express the full repertoire of TLRs and initiate innate responses following activation, we performed the present study. Our data showed that, in addition to TLR4, 661W cells express TLR1, 2, 3, 5, 6, 7, 8, and 9, and that they are all functional. These findings indicate the versatile nature of photoreceptors in responding to a wide variety of infectious stimuli and their active role in retinal innate immunity.

## Materials And Methods

### Bacterial Strain and Reagents

*S*. *aureus* (strain RN6390) [11,12,13,14] was maintained in tryptic soy broth (TSB; Sigma-Aldrich, St. Louis, MO). Bacterial lipopeptide Pam3Cys-Ser-(Lys)4 hydrochloride (Pam3Cys, TLR1/2 agonist), polyI:C (TLR3 agonist), Lipoploysacchride (LPS, TLR4 agonist), flagellin (TLR5 agonist), PolydT (TLR7/8 agonist), and ODN (TLR9 agonist) were purchased from InvivoGen (San Diego, CA). Antibodies against p-ERK, ERK, phospho-p38, p38, IkB-α, and TLR 3, 4, 5, 7, and 9 were purchased from Santa Cruz Biotechnology Inc. (CA, USA). Anti-phospho-IkB-α and anti-TLR2 antibodies were purchased from Cell Signaling Technology (Beverly, MA). A mouse monoclonal anti-β-actin antibody was purchased from Sigma (St. Louis, MO). Secondary horseradish peroxidase (HRP)-conjugated anti-mouse or anti-rabbit IgG antibodies were purchased from Bio-Rad (Hercules, CA).

### 661W Cell Culture

Mouse cone photoreceptor cell line 661W was provided by Dr. Muayyad Al-Ubaidi (Department of Cell Biology, University of Oklahoma Health Sciences Center, Oklahoma City, OK) [15,16]. The 661W cell line was maintained in Dulbecco’s modified Eagle’s medium (DMEM) supplemented with 10% fetal bovine serum (FBS), 10μg/ml L-glutamine, 1% Penicillin & Streptomycin, 40μg/L hydrocortisone, 40μg/L progesterone, 32mg/L putrescine, and 40μl/L β-mercaptoethanol. Cells were grown at 37°C with 5% CO2. When appropriate, cells were grown in serum and antibiotic free DMEM prior to challenge.

### RNA Extraction and PCR Analysis for TLRs

Total RNA was extracted from the 661W cells using TRIzol reagent, as per the manufacturer’s instruction (Invitrogen, Carlsbad, CA). cDNA was synthesized using 1 μg of total RNA using a Maxima first strand cDNA synthesis kit, as per the manufacturer’s instructions (Thermo scientific, Rockford, IL). The cDNA was amplified using TLR (TLR1–9) gene-specific PCR primers synthesized from Integrated DNA Technologies (Coralville, IA, USA) [11] using a PCR condition of initial denaturation at 94°C for 5 min., followed by 35 cycles of denaturation (94°C, 45 sec.), annealing (50°C, 1 min.), and extension (72°C, 2 min.), with a final extension at 72°C for 10 min. The PCR product and the internal control (GAPDH) were subjected to electrophoresis on 1.5% agarose gel containing 0.5 μg/ml ethidium bromide. Stained gels were captured using a digital camera (EDAS 290 system, Eastman Kodak, Rochester, NY).

### Western Blotting

661W cells were challenged with *S*. *aureus* RN6390 and different TLR agonists, viz. Pam3CSK4 (TLR1/2), PolyI:C (TLR3), LPS (TLR4), Flagellin (TLR5), Poly(dT) (TLR7/8), and ODN (TLR9), for 8h. Following incubation, 661W cells were lysed with radio-immunoprecipitation assay (RIPA) buffer [150 mM NaCl, 100 mM Tris-HCl (pH 7.5), 1% deoxycholate, 0.1% sodium dodecyl sulfate (SDS), 1% Triton X-100, 50 mM NaF, 100 mM sodium pyrophosphate, and 3.5 mM sodium orthovanadate]. A protease inhibitor cocktail containing aprotinin, pepstatin A, leupeptin, and antipain (1 mg/ml each), and 0.1 M phenylmethylsulfonyl fluoride (Sigma-Aldrich) was added to the RIPA buffer before use (1 μl/ml). The total protein concentration of the cell lysate was determined using a Micro BCA protein assay kit (Thermoscientific, Rockford, IL). Total protein samples (30 μg) were resolved on SDS-PAGE in Tris-glycine-SDS buffer (25 mM Tris, 250 mM glycine, and 0.1% SDS) and electro-blotted onto a polyvinylidene fluoride (PVDF) membrane (Millipore, Billerica, MA). After blocking for 1h in 5% MPBST (phosphate buffered saline (PBS) containing 0.05% Tween 20 and 5% nonfat milk), the blots were probed with primary antibodies (1:1000) overnight at 4°C. The membranes were washed three times with PBST (PBS containing 0.05% Tween 20) and incubated with horseradish peroxidase (HRP) conjugated secondary antibodies (BioRad) diluted in 5% MPBST at RT for 1h. Protein bands were visualized with Supersignal West Femto Chemiluminescent Substrate (Thermo scientific, Rockford, IL).

### Enzyme-Linked Immunosorbent Assay (ELISA)

661W cells (1x10^6^/well) were cultured in a 6 well dish. Following overnight growth factor starvation, cells were challenged with various TLR agonists (10 μg/ml) or with *S*. *aureus* (MOI 20:1). In blocking experiments, 661W cells were incubated with 10 μg/ml of TLR2-neutralizing antibody (Abcam, Cambridge, MA) for 1 h at 37°C prior to stimulation with *S*. *aureus* or Pam3. Following incubation, the culture supernatants were collected and the levels of IL-1β, IL-6, MIP2, and KC were determined by ELISA. ELISA was performed as per the manufacturer’s instructions [BD biosciences, San Diego, CA (IL-6 & IL1β) and R & D systems, Minneapolis, MN (MIP2 & KC)].

### Immunohistochemistry

661W cells were cultured on four well glass chamber slides (Fisher Scientific, Rochester, NY) and stimulated with *S*. *aureus* and different TLR agonists for 8h. The cells were washed three times with PBS and fixed for 15 min. in 4% paraformaldehyde in PBS. After washing, the cells were permeabilized for 10 min. with an ethanol:acetic acid mixture (2:1) at -20°C and washed once more. The fixed cells were then blocked in 1% (w/v) BSA for 1h at room temperature, followed by incubation with primary antibodies (1:100 dilution) overnight at 4°C. Following removal of the primary antibodies, the cells were washed extensively with PBS and incubated for 1h with specific fluorescein isothiocyanate (FITC)-conjugated secondary antibodies (1:200 dilution) at room temperature. Finally, the cells were again extensively washed with PBS and the slides were mounted in Vectashield anti-fade mounting medium (Vector Laboratories) and visualized using an Eclipse 90i fluorescence microscope (Nikon, Melville, NY).

## Results

### The Cone Photoreceptor Cell Line 661W Constitutively Expresses TLRs

**Fig 1:**
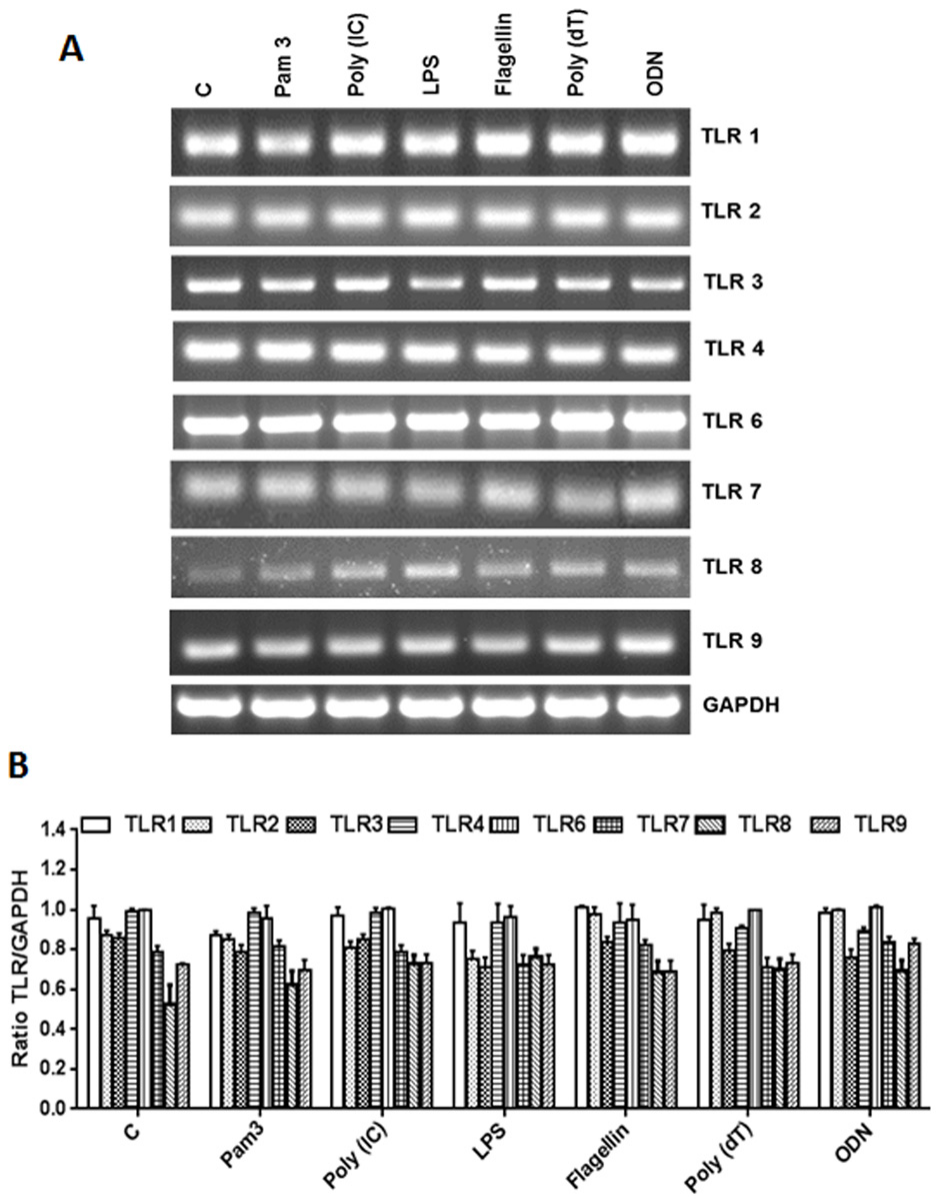
RT-PCR analysis of TLR expression in 661W cells. 661W cells were either left untreated (control) or challenged with the indicated TLR ligands: Pam3CSK4 (10 μg/ml), Poly(IC) (10 μM), LPS (10 μg/ml), Flagellin (250 ng/ml), Poly(dT) (10 μM), or ODN (10 μg/ml) for 8h. Total RNA was extracted, reverse transcribed, and subjected to semi-quantitative RT-PCR using primers for specific TLRs, with glyceraldehyde 3-phosphate dehydrogenase (GAPDH) as the control (A). Band intensity was quantified by densitometric analysis using Image J analysis software (NIH) and presented as the relative band intensity of TLRs vs. GAPDH (B). Data points and error bars represent mean ± SD from two independent experiments.

**Fig 2:**
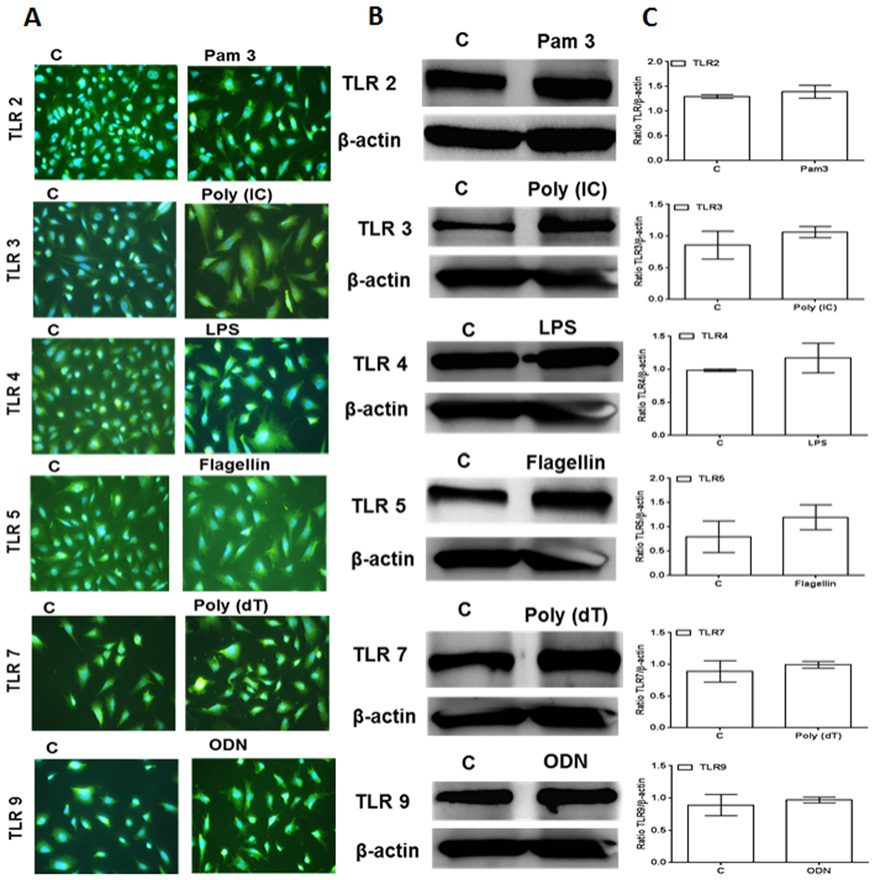
Western blot and IHC Analysis of TLR Expression in 661W cells. 661W cells were either left untreated or challenged with the indicated TLR ligands (as described in Fig. 1 legend) for 8h. Immunostaining was performed using specific antibodies to access TLR expression (A). In a separate experiment, cells were lysed using RIPA buffer and analyzed for TLRs expression by Western blot analysis using specific anti-TLR antibodies, with β-actin as a loading control (B). Band intensity was quantified by using Image J and presented as the relative band intensity of TLR vs. β-actin (C). Data points and error bars represent mean ± SD from two independent experiments.

TLRs play an important role in the initiation of early innate immune responses in various ocular tissues, including the retina [2,11]. However, the role of TLRs in the generation of innate responses by retinal neurons, such as the photoreceptor cells, is not fully understood at this time. First, we assessed the expression of TLRs on photoreceptor cells using the cone photoreceptor cell line 661W [17]. As shown in Fig. 1A, 661W cells were found to express transcripts of TLRs 1 through 9. However, a semi-quantitative analysis revealed no significant differences in the TLR levels of control (unstimulated) vs. TLR ligand-challenged cells (Fig. 1B). The expression of TLRs at the protein level was assessed by immunostaining, which showed no significant differences in immunofluorescence intensity of TLRs in control (unstimulated) versus TLR-ligand stimulated cells (Fig. 2A). Similarly, the western blot analysis, although revealing slight increases in the expression of TLR2, 3, 4, 5, and 7 following challenge with their respective ligands (Fig. 2B), these differences did not reach statistical significance upon quantification (Fig. 2C). Taken together, our results indicate that 661W cells constitutively express TLRs irrespective of TLR agonist challenge.

### TLR Ligands Induce the Activation of NF-kB, p38, and ERK Signaling

**Fig 3:**
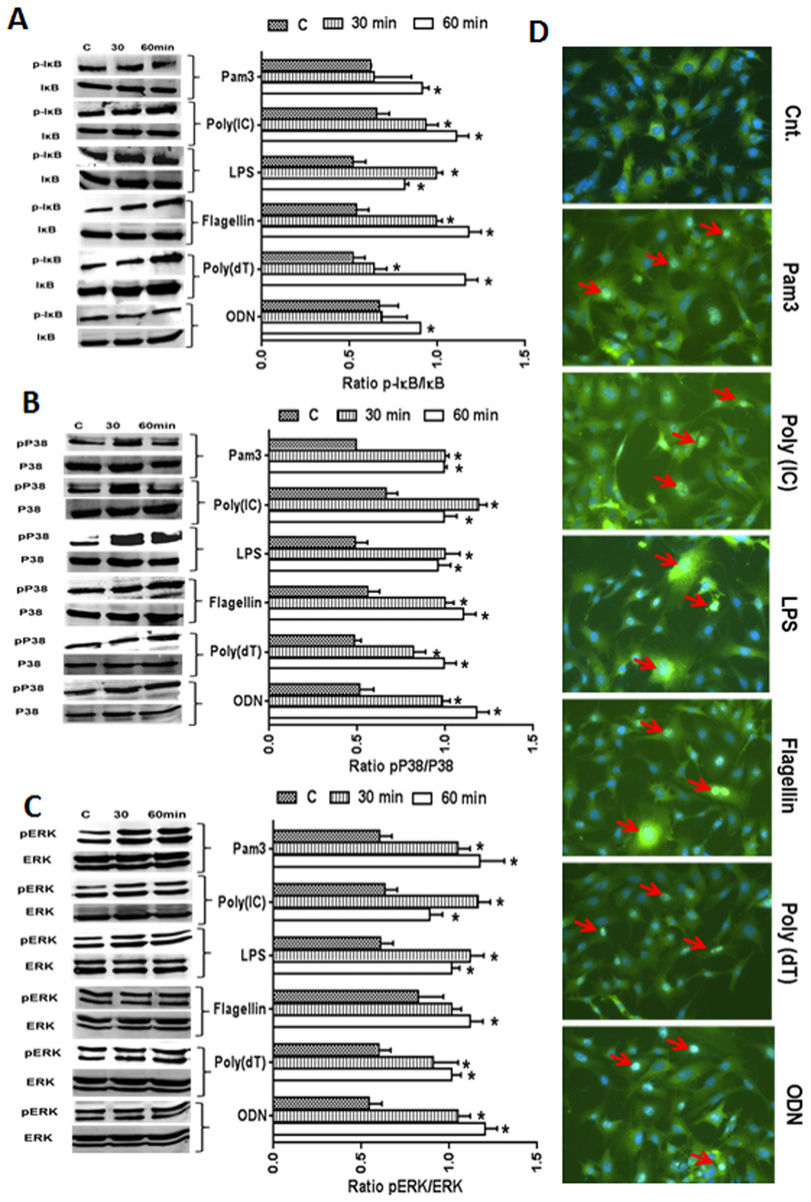
Activation of TLR-downstream (NF-kB and MAPK) signaling following TLR agonist stimulation. In order to assess the activation of IkB, p38, and ERK signaling following stimulation with TLR agonists for 30 and 60 min., 661W cells were lysed in RIPA buffer and analyzed by Western blot analysis using antibodies against phospho-IkB (p-IkB), p-p38, and p-ERK. Antibodies against ERK, p38, and IkB were used to detect total protein levels (A-C). Band intensity was quantified using Image J and presented as the relative band intensity of the phosphorylated form vs. the total form for the respective proteins (A-C). To detect the nuclear translocation of NF-kB (p65), 661W cells were challenged for 60 min. with various TLR ligands. Cells were then fixed, permeablized, and immune-stained with antibodies against p65, a subunit of NF-kB (D). Statistical analysis was performed using one-way ANOVA (*, p<0.05), for comparisons of control versus 30 and 60 min. stimulation.

The expression of TLRs suggests that the photoreceptor cells would be responsive to TLR ligands. To test this hypothesis, we measured the ability of TLR 2, 3, 4, 5, 7, and 9 ligands to activate NF-kB and MPAKs (p38 and ERK) signaling in 661W cells. As shown in Fig. 3, 661 W cells challenged with TLR agonists (10 μg/ml) showed activation of the IkB, p38, and ERK pathways, as evidenced by increased levels of phosphorylated IkB (Fig. 3A), p38 (Fig. 3B), and ERK (Fig. 3C) at both the 30 and 60 min. time points, albeit with higher phosphorylation at 60 min. NF-kB activation was further confirmed by its translocation from the cytoplasm to the nucleus by immunostaining for p65, a functional subunit of NF-kB. While no nuclear staining of p65 was observed in control (unstimulated) cells, all TLR ligand-treated cells exhibited a nuclear staining pattern of p65, suggesting that NF-kB is activated in these cells (Fig. 3D). This finding demonstrates that TLR-mediated intracellular signalling pathways are operational in 661W cells, an indication of functional TLRs.

### TLR Ligand-Stimulated 661W Cells Produce Inflammatory Mediators

**Fig 4:**
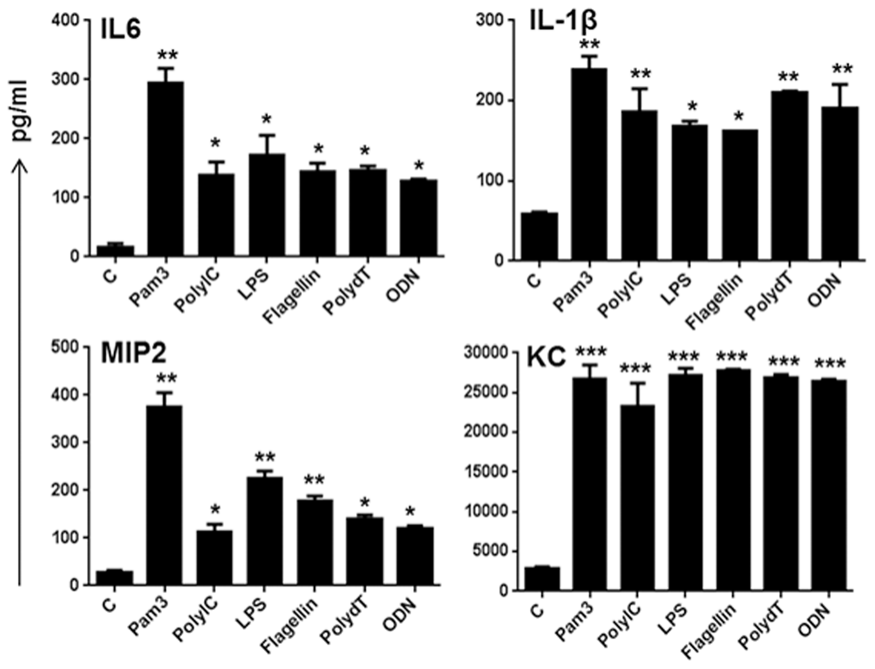
Effect of TLR agonists on secretion of inflammatory mediators in 661W cells. 661W cells were challenged with the indicated TLR ligands for 8h and the secretion of inflammatory mediators was measured in conditioned media by ELISA. Data points and error bars represents mean ± SD of duplicates from three independent experiments. Statistical analysis was performed using one-way ANOVA (*, p<0.05; **, p<0.005; ***, p<0.005), for comparisons of control versus stimulated cells.

To assess the biological relevance of induced NF-kB, p38, and ERK activation, we measured the effect of TLR ligands on pro-inflammatory cytokine production (secretion) by ELISA. Our time-course studies (data not shown) showed a significant accumulation of inflammatory cytokines/chemokines at an 8h time point. Among the various TLR ligands, the TLR2 ligand Pam3Cys was shown to induce higher levels of all inflammatory mediators (IL-6, IL-1β, MIP2, and KC), whereas the response of other TLR ligands varied (Fig. 4). Similarly, among the tested cytokines/chemokines, KC levels were ~100 times more. Taken together, these results indicate that photoreceptor cells are responsive to TLR ligands and generate inflammatory response.

### 661W Cells Elicit Innate Responses Following Live Bacterial Challenge

**Fig 5:**
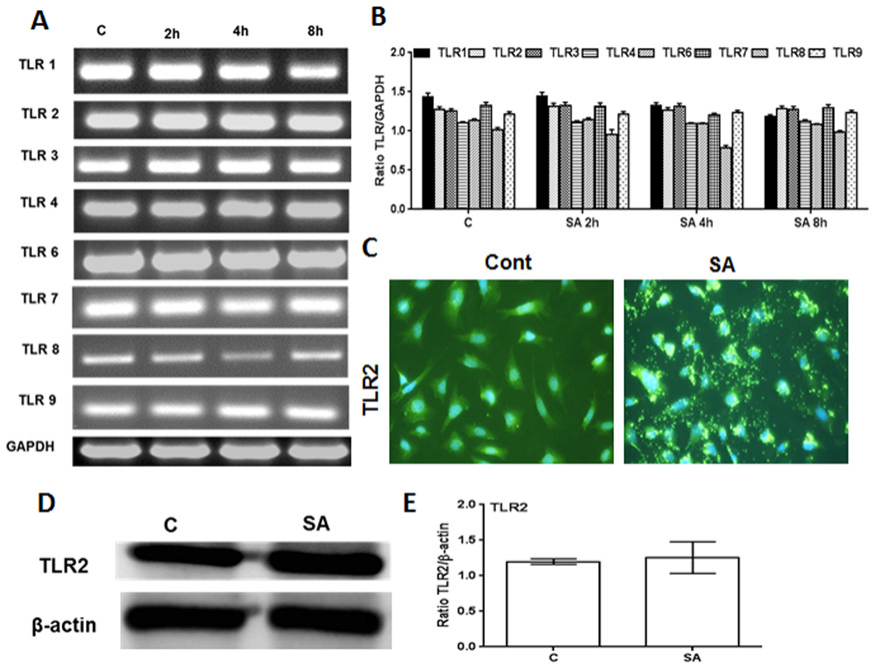
Modulation of TLR expression in 661 W cells following S. aureus challenge. 661W cells were stimulated with *S*. *aureus* (MOI 20:1) for the indicated time points. Total RNA was extracted, reverse transcribed, and subjected to semi-quantitative PCR for various TLRs (A). Band intensity was quantified by densitometric analysis using image J analysis software (NIH) and presented as the relative band intensity of TLRs vs. GAPDH (B). In a separate experiment, 661W cells were challenged with *S*. *aureus* (MOI 20:1) for 8 h and Immunostaining was performed using TLR2 specific antibody (C). TLR2 expression was further confirmed by Western blot analysis (D) and band intensity was quantified using Image J (E).

**Fig 6:**
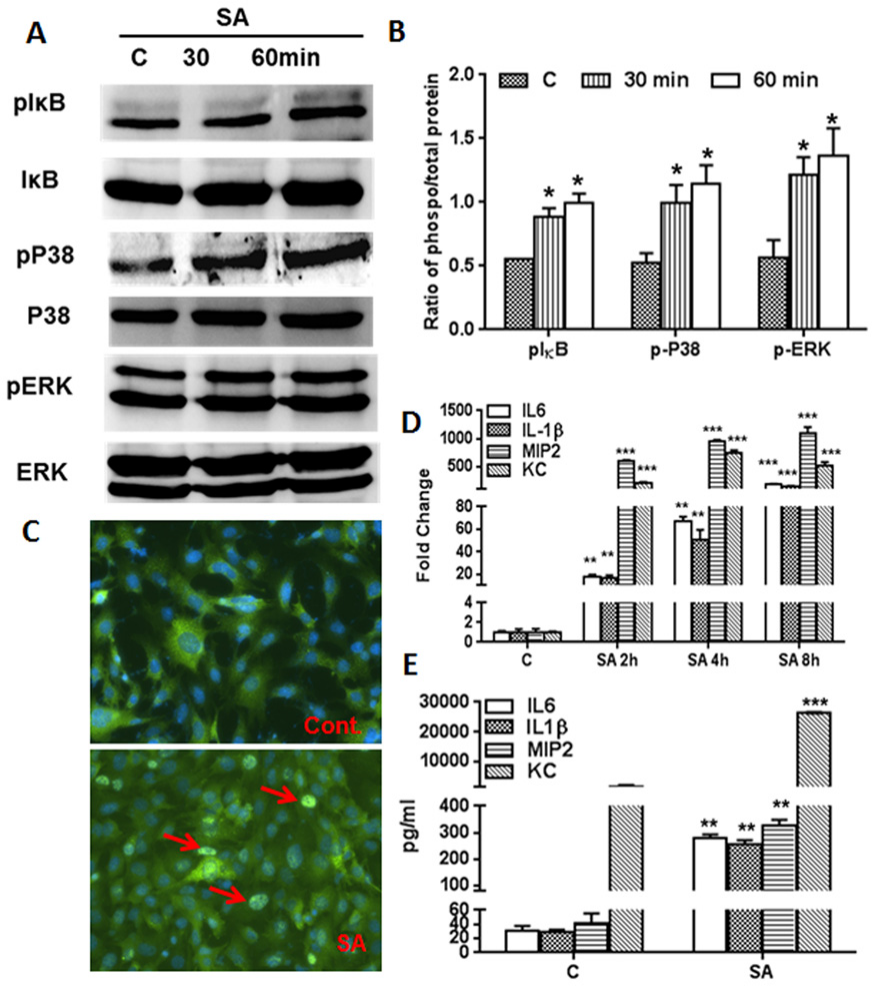
S. aureus-triggered innate responses in 661W cells. 661W cells were challenged with *S*. *aureus* (MOI 20:1) for the indicated time points and Western blot was performed for IkB, p38, and ERK using phosphorylated and non-phosphorylated antibodies (A). Band intensity was quantified using Image J (B). Immunostaining was performed to monitor the localization of NF-kB to the nucleus (C). qRT-PCR was performed for the quantification of induced mRNA expression of inflammatory mediators (IL-6, IL-1β, CXCL1, and CXCL2) and the results were expressed as relative fold changes with respect to a GAPDH control (D). The level of secretary cytokines/chemokines was measured by ELISA (E). Data points and error bars represents mean ± SD of triplicates from two independent experiments. Statistical analysis was performed using one-way ANOVA (*, p<0.05; **, p<0.005; ***, p<0.0005), for comparisons of control versus stimulated cells.

The forgoing results indicate that 661W cells constitutively express TLRs and that their engagement with their respective ligands evokes inflammatory response. To determine whether photoreceptors respond to live infection, we investigated the innate response of 661W cells towards *S*. *aureus* (strain RN6390), the leading cause of severe retinal damage in endophthalmitis. First, we performed a time-course study to assess the modulation of TLR expression in 661W cells following *S*. *aureus* challenge (Fig. 5A). Similar to TLR ligand treatment (Fig. 1), *S*. *aureus* infection did not significantly alter the expression of TLRs (Fig. 5B). Since TLR2 has been shown to be the major receptor for the recognition of Gram-positive bacteria, including *S*. *aureus*, we determined the expression of TLR2 by immunostaining and western blot analysis and observed a constitutive expression of TLR2 protein (Fig. 5C & E). However, *S*. *aureus*-infected 661W cells showed activation of TLR-downstream signaling pathways (Fig. 6A & B), including the translocation of NF-kB from the cytoplasm to the nucleus in 661W cells (Fig. 6C). Moreover, *S*. *aureus* challenged 661W cells exhibited time-dependent mRNA expression (Fig. 6D) and secretion (Fig. 6E) of inflammatory cytokines (IL-1β and IL-6) and chemokines (KC and MIP2), as assessed by qRT-PCR and ELISA, respectively.

### *S*. *aureus* Triggered Inflammatory Response in 661W Cells is TLR2-Independent

**Fig 7:**
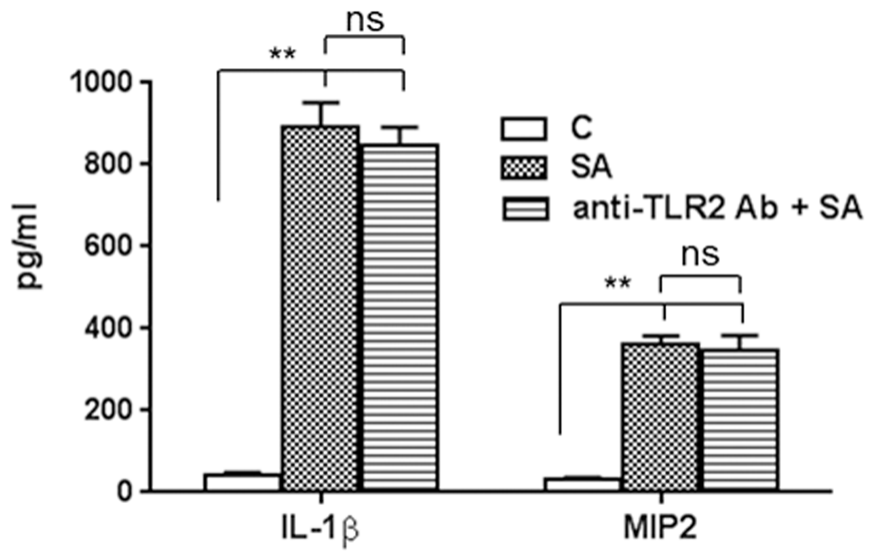
Effect of TLR2 neutralization on inflammatory responses towards S. aureus. 661W cells were challenged with SA for 8 h following neutralization of TLR2 using anti-TLR2 neutralizing antibody. The expression of secretory cytokines and chemokines were measured by ELISA in the conditioned medium.Statistical analysis was performed using one-way ANOVA (**, p<0.005; ns, not significant).

We previously showed that retinal glial cells initiate an innate response towards *S*. *aureus* via TLR2 and that the neutralization/inhibition of TLR2 signaling attenuated these innate responses [18,19,20]. To determine whether 661W cells also generate an innate immune response through TLR2, we blocked the TLR2 receptor using anti-TLR2 neutralizing antibody. These inhibition studies revealed that 661W cells were still able to respond to *S*. *aureus* challenge without functional TLR2, as evidenced by similar levels of IL-1β and MIP2 in control cells (no blocking) versus cells incubated with anti-TLR2 antibody (Fig. 7). In contrast, blocking TLR2 inhibited the Pam3Cys-mediated production of inflammatory cytokines (data not shown). These results indicate the existence of TLR2-independent mechanisms in 661W cells to respond to live *S*. *aureus* challenge.

## Discussion

Innate immunity provides the first line of defence against invading pathogens [3,4]. In the retina, innate immune responses are initiated by the recognition of pathogens by TLRs expressed on retinal glial cells [7,11,19,21]. In turn, the activated glial cells produce anti-microbial and pro-inflammatory mediators to recruit professional innate immune cells, such as PMNs, to limit bacterial growth and destroy the pathogens [12,22]. In this study, we demonstrate that TLR expression is not confined to the glial cells of the retina; rather, they are expressed on retinal neurons, such as the photoreceptor cells. Moreover, our data showed that the expressed TLRs are functional, as evidenced by the activation of TLR-mediated downstream signaling (NF-kB, p38, and ERK) and the production of inflammatory cytokines and chemokines. Furthermore, photoreceptor cells were found to elicit inflammatory responses following TLR ligand or live bacterial (*S*. *aureus*) challenge, implicating their role in providing retinal innate defense under infectious conditions, such as bacterial endophthalmitis.

In contrast to mucosal surfaces, the retina resides in a sterile environment. As a result, one might expect that retinal cells express TLRs at the basal level in the resting state, and, upon pathogen interaction, TLR expression would be upregulated, as seen in classical innate immune cells. This was certainly the case with retinal glial cells, which exhibited an increased expression of TLR2 following *S*. *aureus* challenge [18,19,21]. Although our data showed the expression of all TLRs in 661W cells, neither their mRNA nor protein levels changed following stimulation with TLR ligand, indicating a constitutive expression pattern of the TLRs. These finding are consistent with Tu *et*. *al*. [23], who reported the constitutive expression of TLR4. However, these findings raise important questions: why do photoreceptor cells express TLRs and what is their biological significance in non-infectious conditions. An increasing number of studies have highlighted the importance of TLRs beyond their traditional role in response to pathogens [24]. For example, they have been implicated in regulating sterile inflammation in various tissues, including the retina [25,26,27]. TLRs have also been shown to orchestrate the innate immune response to trauma by recognizing danger-associated molecular patterns (DAMPs) that are released from injured tissues [28,29]. Indeed, several DAMPs, including HMGB1 and heat shock proteins (HSP60, HSP70), are released upon tissue injury and are known ligands for several TLRs. [30,31,32,33]. We have also observed higher levels of HMGB1 in *S*. *aureus*-infected retinal tissue (unpublished data). Thus, we propose that, in the absence of microbial challenge, photoreceptor TLRs can detect intrinsic danger signals to modulate photoreceptor cell survival and function.

It is now well established that, following recognition of PAMPs or DAMPs, the downstream TLR signaling cascade leads to the phosphorylation of the inhibitor of NF-kB (IkB)–kinase complex (IKK complex) [7,34]. The phosphorylation of IkB results in the degradation of IkB, allowing the translocation of NF-kB to the nucleus to initiate the transcription of inflammatory genes [35]. Similarly, our data showed the activation of NF-kB in 661W cells following stimulation with TLR ligand. In addition to NF-kB, there is also activation of tumor necrosis factor receptor associated factor 6 (TRAF6), a downstream adaptor molecule that can activate the MAPK signaling pathways(such as JNK, p38, and ERK), which leads to a further increase in the transcription and translation of inflammatory mediators, including chemokines and cytokines, such as IL-8, MIP2, KC, TNF-α, IL-1β, IL-6, and IL-8, as well as reactive oxygen/nitrogen species (ROS/RNS). Knowing this, it was not surprising to observe the activation of ERK and p38 signaling and the secretion of inflammatory mediators in 661W cells stimulated with specific TLR agonists. However, the generation of classical innate responses following live *S*. *aureus* challenge was intriguing, considering the non-immune nature of photoreceptor cells. Earlier, we showed that retinal glial cells initiate innate responses towards *S*. *aureus* via TLR2 signaling [11,12,36]. However, this does not seem to be the case with 661W cells, suggesting the existence of TLR2-independent mechanisms, an intriguing topic which requires further investigation. Regardless, these findings indicate that photoreceptor cells could be a source of inflammatory cytokines and chemokines during retinal infection and may contribute to the pathogenesis of infectious endophthalmitis.

### Conclusions

In summary, as photoreceptor cell death plays a crucial role in the pathogenesis of retinal infections, pathogen recognition mechanisms must have been evolved for their defence and survival. Here, we have described TLR-mediated mechanisms in cone photoreceptors that aid in retinal innate immunity. Our data clearly demonstrate that photoreceptors have the ability to generate innate responses following challenge with TLR ligand and live bacteria. However, further investigation is needed to validate these findings in an *in vivo* model of retinal infection.
